# Modeling perinatal brain injury with human brain organoids: from pathophysiology to treatment development

**DOI:** 10.1186/s13287-026-05228-6

**Published:** 2026-08-05

**Authors:** Zahra Dehghani, Daniel Surbek, Marianne Joerger-Messerli, Andreina Schoeberlein

**Affiliations:** 1 Department of Obstetrics and Feto-maternal Medicine, lnselspital, Bern University Hospital, University of Bern, Bern, Switzerland; 2 Department for BioMedical Research (DBMR), University of Bern, Bern, Switzerland; 3 Graduate School for Cellular and Biomedical Sciences (GCB), University of Bern, Bern, Switzerland

**Keywords:** Perinatal brain injury, Brain organoid, Preterm birth, Neurodevelopmental disorders, Organoid-based drug development

## Abstract

Perinatal brain injury (PBI) is a leading cause of childhood morbidity and mortality, often resulting in long-term neurological deficits. Despite its relatively high prevalence, PBI lacks reliable biomarkers for early detection and effective therapeutic approaches. The etiology of PBI is multifactorial and includes not only preterm birth but also hypoxia-ischemia, infection, and inflammation. While animal models and two-dimensional cell cultures have contributed to our understanding of PBI pathophysiology as well as to the development of novel treatment strategies, they fail to fully capture the complexity of injury in the developing human brain. Human brain organoids have recently emerged as transformative platforms recapitulating key features of fetal-neonatal brain development, including relevant cell types, gene expression patterns, cytoarchitecture, and functional electrophysiological properties. This model therefore provides opportunities to investigate the mechanisms underlying perinatal insults and serves as a promising tool for novel treatments such as stem cell-based therapy, drug discovery and screening, helping to bridge the translational gap between preclinical studies in animal models and clinical applications. Recent innovations, including the development of vascularization strategies, the incorporation of glial cells, brain organoid-on-chip technologies, and assembloids, have further increased the physiological relevance of these methods. This review highlights recent advances in organoid-based models of PBI and highlights their potential and challenges as next-generation tools for mechanistic studies and therapeutic innovations.

## Introduction to perinatal brain injury and its major etiologies

Perinatal brain injury (PBI) is associated with significant neonatal morbidity, increased mortality, and long-term neurological deficits, particularly in very preterm-born infants (before gestational week (GW) 32) [1]. Despite significant improvements in obstetric and neonatal care, the rate of severe neurodevelopmental disability remains high among survivors [2–5]. The primary etiologies of PBI include hypoxia alone or combined with ischemia, as well as maternal, fetal or neonatal infection and inflammation [1]. These insults occur during a highly dynamic period of brain development between GW 24–40, in both preterm and term-born infants, when complex cellular and developmental processes are actively unfolding across multiple cellular populations, including preoligodendrocytes (pre-OLs), microglia, subplate neurons, and developing axons. Concurrently, key developmental and proliferative regions such as the dorsal subventricular zone, ganglionic eminence, cortex, and cerebellum are undergoing active maturation [1]. During this developmental window, these regions and cell populations are particularly vulnerable to endogenous and exogenous insults, including ischemia, inflammation, excitotoxicity, oxidative stress, and exposure to certain drugs, hormones, or neurotoxins, leading to macro- or microscopic neuropathological lesions in white and grey matter, and ultimately contributing to long-term neurodevelopmental sequelae, including behavioral abnormalities and motor and sensory deficits in survivors [1, 6]. The gap in understanding how maternal and fetal exposures affect injury mechanisms underscores the challenge in developing effective therapies. A wide range of neuroprotective strategies has been investigated to mitigate the mechanisms underlying PBI. However, most of these remain in early translational stages. A key factor limiting clinical translation is the interspecies differences between current experimental research models and humans. The lissencephalic rodents have simpler brain structures and do not adequately replicate the human-specific features of corticogenesis and white matter vulnerability. Although gyrencephalic nonhuman primates offer a closer approximation [7], ethical and financial constraints limit their use. Access to human fetal brain tissue for experimental research is extremely limited and is accompanied by considerable ethical concerns and methodological limitations [8–10]. Two-dimensional (2D) in vitro models lack the cellular diversity, interconnectivity, and cytoarchitectural properties of the developing brain, as well as an accurate representation of disease pathology [11].

Thus, establishing a PBI research model that more closely reflects the physiology and function of the human fetal brain has recently directed scientists to use human pluripotent stem cell (PSC)-derived brain organoids [12], recapitulating the cellular composition, cytoarchitecture, human-specific neurodevelopmental milestones, and functional electrophysiological activity of the human neocortex while maintaining experimental scalability and tractability [13]. In summary, despite clear neuropathological evidence of PBI, there are no widely accepted clinical biomarkers or established therapies for prevention and treatment. This highlights the urgent need for improved models that can better simulate human brain development and injury mechanisms.

## Overview of brain organoids

### Advantages of using brain organoids

Brain organoids recapitulate several important aspects of the developing human brain that make them ideal for studying PBI. First, they are potentially able to include almost all cell types that exist in the fetal brain, including neural progenitors, excitatory neurons, interneurons, astrocytes, and even vascular cells [14], microglia [15], and oligodendrocytes [16–19], enabling cell‒cell communication between different cell types in a 3D environment.

Second, they have morphological and transcriptional similarities to the fetal brain in several developmental stages, especially stages that are more susceptible to PBI. These brain organoids can be generated either as unguided whole-brain models, commonly referred to as cerebral organoids (COs) [12], or as guided region-specific organoids, including forebrain (such as cortical and ventral forebrain organoids), midbrain, hindbrain, hypothalamic, hippocampal, cerebellar, and spinal cord organoids, depending on the differentiation and patterning strategy employed [20–27]. The 3D structures of brain organoids facilitate the proliferation, migration, and differentiation of different cell types. Unguided cerebral organoid protocols can spontaneously generate cortical-like regions exhibiting rudimentary laminar organization, resembling that of the developing human cortex in vivo, from ventricular zones (VZ) containing neural progenitors to the subventricular zone (SVZ), intermediate zone (IZ), and cortical plate-like layer with more mature neurons [12].

Third, these organoids are electrically active and can even reproduce complex functional networks and disease phenotypes. They exhibit spontaneous neuronal firing, synaptic connectivity, and oscillatory network activity that resemble those of the developing human brain, facilitating the investigation of functional maturation, neural circuit formation, and network-level abnormalities observed in neurodevelopmental disorders [22, 23, 28–30].

### Relevance to prenatal developmental stages

*In utero*, human nervous system development begins with neurulation to form the neural tube, proceeds through expansion of neural stem/progenitor pools, transitions to sequential waves of neurogenesis followed by gliogenesis, and culminates in the assembly and maturation of functional neuronal circuits [13]. Protocols for brain organoid generation from PSCs typically begin by aggregating PSCs into 3D embryoid bodies (EBs), resembling pregastrulation stages that permit early fate decisions. Neural induction then directs EBs toward a neural lineage, commonly via dual SMAD inhibition (blocking transforming growth factor-beta, TGF-β/bone morphogenetic protein, BMP signaling), to promote neuroectodermal fate, in which cells upregulate early neural markers. The resulting neuroepithelial domains expand and organize into a pseudostratified, apicobasally polarized epithelium reminiscent of the early neural tube. Embedding aggregates in an extracellular matrix stabilizes polarity, supports neural rosette formation, and fosters further radial tissue organization [12]. With subsequent maturation, neurogenesis initiates and proceeds in an inside-out sequence analogous to fetal cortical development. Radial glial cells (RGs) function as neural stem/progenitor cells, generating intermediate progenitors and neurons that migrate along RG fibers to establish cortical-like lamination. Prolonged culture supports further differentiation, including the generation of astrocytes, oligodendrocyte precursors, and synaptically active neuronal networks [12, 31, 32].

**Fig. 1:**
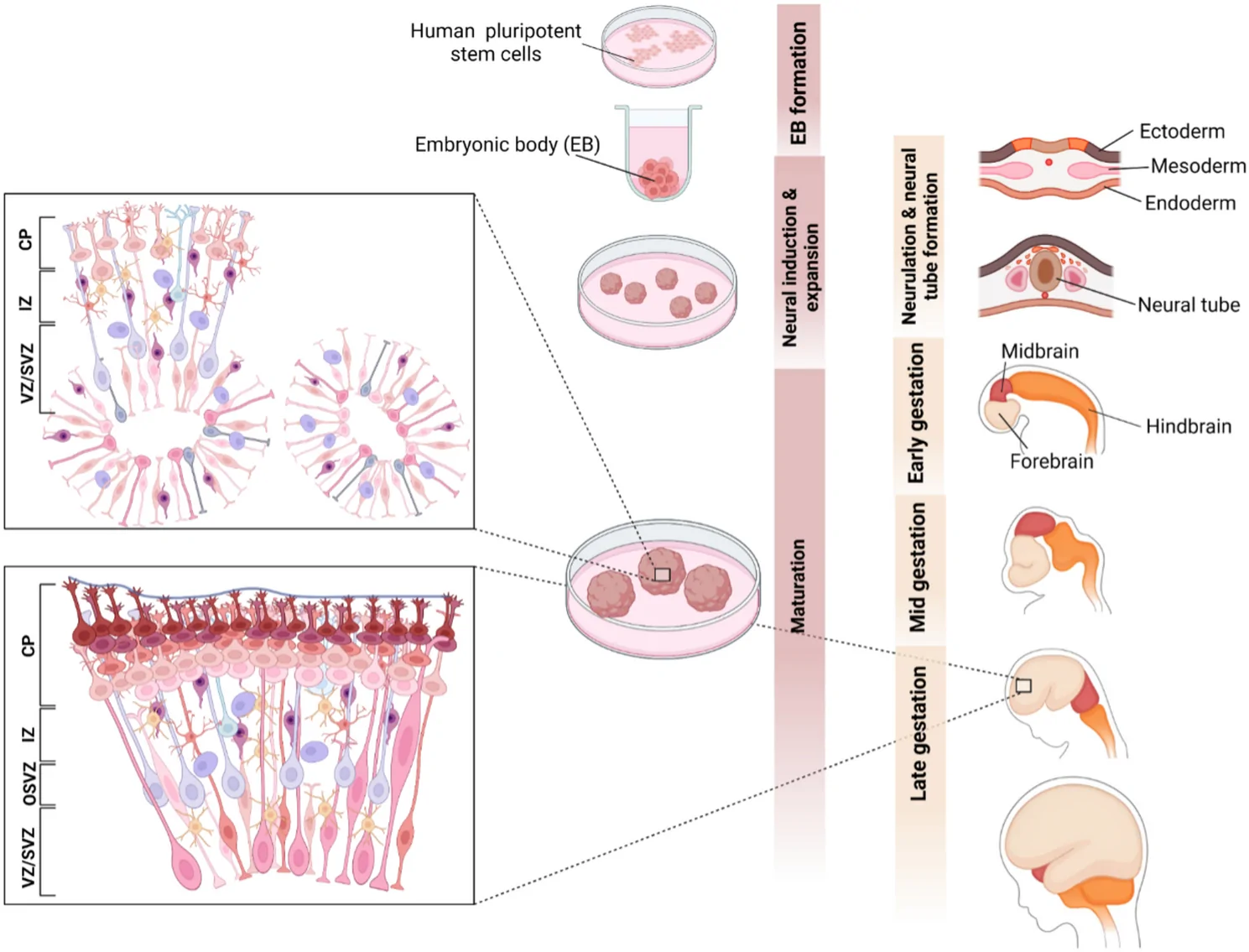
Developmental trajectory of brain organoids recapitulating human brain development. *In utero*, neural development begins with neural induction from the ectoderm, followed by neural tube formation and regionalization into the forebrain, midbrain, and hindbrain. Cerebral organoid protocols mimic these steps by aggregating pluripotent stem cells into 3D embryoid bodies (EBs), directing them toward a neuroectodermal fate, and generating neuroepithelial rosettes that resemble the early neural tube. With maturation, organoids undergo inside-out neurogenesis and establish layered structures similar to those of the fetal cortex, with layers including the ventricular and subventricular zones (VZ/SVZ), outer subventricular zone (oSVZ), intermediate zone (IZ), and six layers of the cortical plate (CP), thereby reproducing the cellular diversity and laminar organization of the developing human brain. Created in https://BioRender.com

To benchmark in vitro models against in vivo human corticogenesis, multiple studies have applied transcriptomic profiling to neocortical cultures using microarrays [22, 33], RNA sequencing [20, 33, 34] or single-cell RNA sequencing [35] and aligned these datasets to reference atlases of the developing human brain. These methods consistently indicate that in vitro systems recapitulate early in vivo developmental programs, from ca. GW 8–10 through the late midfetal period (GW 19–24), supporting the conclusion that early human neocortical development can be modeled with high fidelity via in vitro organoid systems (Fig. 1). In addition to their cellular composition, cortical organoids exhibit emergent network dynamics that resemble the electrographic patterns observed in preterm infants at approximately 28 GW [29]. Furthermore, evidence from genome-wide assessments of the epigenetic clock, transcriptomic profiling, and RNA editing has shown that human cortical organoids can even reach early postnatal maturation features within 250–300 days in vitro [36]. This opens the door not only to studying perinatal brain injury mechanisms but also to a robust platform for the development of drugs and other therapeutics, with the potential to address the longstanding translational gap from bench to bedside.

## Modeling perinatal brain insults

### Hypoxia-ischemia

Hypoxic-ischemic (HI) injury represents the leading cause of perinatal brain damage and is associated with a spectrum of neurological impairments, including cerebral palsy, intellectual disability, delayed cognitive development, and behavioral disturbances. The onset of hypoxic-ischemic encephalopathy (HIE) during gestation can arise from a range of complications, such as impaired placental perfusion, preeclampsia, maternal diabetes, intrauterine infections, substance exposure, severe fetal anemia, or pulmonary disease [37, 38]. HIE involves a series of events that extend over hours to days following the initial insult and is characterized by oxidative stress, inflammation, and progressive neuronal death. The underlying drivers are oxygen and glucose deprivation in the developing brain, which trigger a shift to anaerobic metabolism, depletion of adenosine triphosphate (ATP) reserves, and subsequent failure of ATP-dependent Na⁺/K⁺ pumps. While HI injury typically involves combined oxygen and nutrient deprivation, hypoxia or hyperoxia may also occur independently in preterm infants [39, 40]. Perinatal hypoxia can arise either from environmental hypoxia, in which both the mother and fetus are exposed to reduced oxygen availability, or from placental hypoxia, in which placental dysfunction limits fetal oxygen transfer. The contributing factors include high-altitude pregnancy, environmental pollution, preeclampsia, smoking, alcohol exposure, glucocorticoid administration, and severe maternal stress, all of which can compromise uteroplacental perfusion and reduce oxygen delivery to the developing brain. Additionally, hypoxia may occur in preterm infants after birth due to pulmonary immaturity, as the underdeveloped lungs are often unable to adequately support gas exchange immediately after birth, resulting in insufficient oxygen delivery to vulnerable brain tissue [39]. In contrast, hyperoxia is frequently encountered in preterm infants following premature transition from a physiologically low-oxygen intrauterine environment to relatively hyperoxic extrauterine conditions and may be further exacerbated by supplemental oxygen therapy and mechanical ventilation during neonatal intensive care, particularly in infants with immature antioxidant defense systems. Although oxidative stress and inflammatory responses induced by neonatal hyperoxia have been identified as major contributors to the pathogenesis of perinatal brain injury, the precise cellular and molecular mechanisms underlying hyperoxia-induced brain injury remain incompletely understood [41, 42].

Brain organoids provide an advanced in vitro platform for investigating HI injury or oxygen–glucose deprivation (OGD)-based models [43, 44] and hypoxia/hyperoxia exposure models [45], enabling detailed exploration of cellular mechanisms, injury responses, potential biomarkers, and therapeutic interventions. Several studies have examined hypoxic injury under various oxygen concentrations, exposure durations, developmental stages of brain organoids, and postinsult recovery conditions [45–49], as summarized in Table 1. Collectively, these studies demonstrate that hypoxic injury in brain organoids induces strong developmental stage- and cell type-specific effects. Indeed, early-stage organoids show pronounced sensitivity, with hypoxia leading to disruption of cortical organization, apoptosis in the VZ, and subsequent loss of basal progenitors, intermediate progenitor cells (IPs), and outer RGs (oRGs), resulting in long-term deficits in cortical marker expression [46, 47]. In contrast, hypoxic insults at later stages generally produce milder phenotypes, often restricted to IPs, which exhibit reduced proliferative capacity through the activation of stress‒response pathways such as the unfolded protein response (UPR) [45, 48]. Reoxygenation can partially restore proliferation but fails to fully rescue neuronal maturation, particularly after severe hypoxia. Importantly, vascularized CO models (vCOs) exhibit complex, human-specific neurovascular and inflammatory responses to hypoxia, highlighting lineage-specific vulnerabilities and context-dependent neuroprotective mechanisms [50, 51]. Overall, these findings underscore that effective modeling of hypoxic-ischemic pathophysiology in brain organoids requires aligning the insult to vulnerable developmental stages, testing and validating varying oxygen levels, combining hypoxia with glucose deprivation and reoxygenation to mimic ischemia‒reperfusion [48, 52], and incorporating key cellular components such as microglia, oligodendrocyte lineage cells, and vascular elements, which are often neglected in most existing studies [45–47]. It is important to note that oxygen–glucose deprivation (OGD) cannot fully recapitulate hypoxic–ischemic injury that occurs in vivo; in practice, it primarily involves reduced oxygen and glucose availability. In vivo ischemia is considerably more complex and additionally involves impaired delivery of other essential nutrients, including amino acids and lipids, reduced clearance of metabolic waste products, CO₂ accumulation, lactate-associated acidosis, altered perfusion dynamics, and endothelial dysfunction that may contribute to prolonged blood–brain barrier impairment [53]. Therefore, although OGD effectively reproduces key aspects of cellular energy failure and metabolic collapse, it only partially recapitulates the broader pathophysiology of hypoxic–ischemic injury in both 2D culture systems and more advanced 3D brain organoid models. Notably, some studies have employed glucose-free Balanced Salt Solution (BSS)-based media [54] rather than conventional glucose-free culture media [55], which may more closely mimic the metabolic conditions associated with ischemia.

**Table 1:** Summary of insults modeled in human brain organoids

Insult		Insult parameters	Brain organoid type and insult timepoint	Key findings	Refs.
Hypoxia/Ischemia		Hypoxia; 1% O_2_ for 72 h (acute); 8% O₂ for 25 days (chronic)	10-day-old neural organoids	Prolonged hypoxia repressed forebrain, oligodendrocyte, glial, and cortical layering/migration genes	[46]
		Hypoxia; 1% O_2_ for 48 h, then reoxygenation OGD for 48 h; then reoxygenation	84-day-old COs	Disrupted neuronal components; increased proliferation but poor maturation after reoxygenation; severe irreversible damage after OGD	[49]
		Hypoxia; 3% O_2_ for 24 h, then reoxygenation	28-day-old COs	Immediate widespread cell death; delayed effects: oRG and DCX^+^ vulnerability at 7–14 days; SOX2^+^ cells remained stable	[47]
		Hypoxia; 1% O_2_ for 48 h	75-day-old cortical organoids	IPs forced into premature differentiation via unfolded protein response; vRGs unaffected	[45]
		Hypoxia; 1% O_2_ for 48 h, then reoxygenation	60-day-old vascularized COs	Astrocyte precursors arrested; immature GABAergic neurons collapsed; microglia biphasic inflammation; hypoxia memory in nonneural cells	[50]
		Hypoxia; 0.6% O_2_ for 48 h	40-day-old vascularized COs	Complex hypoxic responses vs. nonassembled COs; downregulated cell cycle/axonogenesis; increased inflammatory markers; BMP signaling protected TBR2^+^ IPs	[51]
		OGD; $\:\le\:$1% O_2_ in glucose-free Earle’s balanced salt solution for 8 h	85-day-old COs	Increased cytotoxicity and apoptosis, evidenced by elevated LDH release and Caspase-3 activity; partial rescue following treatment with pro-/anti-apoptotic and neuroprotective compounds	[43]
Maternal/fetal Immune Activation		IL-6 overexpression for 5–10 days	45-day-old dorsal forebrain organoids	Increased radial glia; disrupted cortical layering; overproduction of excitatory neurons; MHC-I upregulation; translation genes downregulated; NR2F1, a regulator of genes involved in synapse organization and cell migration, dysregulated (species-specific, has not been reported in animal models)	[59]
		IFN-γ exposure for 48 h	Microglia-containing COs	Reduced number of SOX2^+^ progenitors (rescued by microglia), reduced number of NEUN^+^ neurons; altered expression of autism-linked genes; microglia had a protective role	[60]
Maternal Fever and Thermal Shock		Periodic heat shock at 40 °C at 09:30 − 10:30 and 15:30 − 16:30	10–14 and 20-24-day-old COs	Decreased size and impaired neural tube development; abnormal Wnt signaling pathway and reduction of G2/M progenitor cells; increased neural differentiation and reduced proliferation	[67]
Viral Infections		Zika Virus exposure	28-day-old COs	Preferential infection of RGs, apoptosis, organoid growth arrest (microcephaly)	[70]
Viral Infections (cont.)		Zika Virus exposure (cont.)	30- and 10-day-old COs	Microcephaly phenotype replicated; TLR3 upregulation, disrupted neurogenesis, depletion of the neural progenitor population	[71]
			14- and 80-day-old forebrain organoids	Preferential infection of SOX2^+^ progenitors, including oRGs; neurons/astrocytes vulnerable at later stages, disrupted VZ	[20]
			3-, and 8-week-old COs	Restricted growth, loss of infected NPCs, and reduced proliferative/neurogenic capacity; innate immune activation; viral particles tropism to AQP1^+^ cells	[72]
			24-day-old COs	AXL knockout alone failed to prevent ZIKV-induced pathology	[74]
			45-day-old forebrain organoids	Reduced proliferation and disrupted adherens junction formation of vRGs	[77]
		CMV exposure	30-day-old COs	Impaired growth; PDGFRα/EGFR suggested as entry receptors; TBR2⁺ IP infection; disrupted cortical-like layering; altered Ca²⁺ signaling and neural network activity	[82]
			On day 0 of CO generation	Localized necrotic areas; enlarged vacuoles and cysts; disrupted cortical architecture; abnormal β-tubulin III expression; fewer cortical structure sites	[83]
			Engineered neural tissues	Upregulated lipid metabolism pathways; radially arranged VZ were not infected first, but rather nestin-, doublecortin-, SOX1-, musashi-, and vimentin-expressing adjacent side population of postmitotic cells	[84]
		HSV-1 exposure	15-day-old COs	Impaired neural differentiation in infected NSCs; dysregulated cortical layer; abnormal microglial activation with increased inflammatory mediators (TNF-α, IL-6, IL-10, IL-4)	[92]
			60-day-old COs	Changed neuronal electrophysiology; activated antisense transcription; aberrant 3′-end formation and poly(A)-tail extension; changed cellular composition in the infected organoids; cell-type-specific dysregulated molecular pathways; features of early neurodegeneration: downregulation of ECM components (e.g., STMN2, TARDBP/TDP43), and upregulation of the AD-related noncoding RNA (BC200, BCYRN1)	[93]
			60-day-old COs	Reduced neuronal activity; induced TNF signaling pathway	[94]
			10-week-old COs	Marked alterations in neuronal morphology and cell‒cell fusion; infection of MAP2⁺ neurons leading to the formation of neuronal syncytia	[90]
		SARS-CoV-2 exposure	15- and 60-day-old COs	Limited infection on day 15; pronounced infection on day 60; preferentially targeted TUJ1⁺ neurons; associated with Tau mislocalization from axon to soma, hyperphosphorylation, and neuronal loss	[108]
			35-day-old COs	Virus directly targets cortical neurons and NPCs	[105]
			9-week-old COs	Metabolic alterations in infected and adjacent neurons; no IFN responses; SOX2⁺ NPCs and TUJ1⁺ neurons susceptible	[104]
Viral Infections (cont.)		SARS-CoV-2 exposure (cont.)	5, 10, 16, and 22-week-old cortical organoids	Predominant infection in astrocytes via DPP4; reactive gliosis-like changes; minimal infection of other cortical cells	[106]
			52-day-old cortical organoids	Neuronal death and impaired synaptogenesis, both rescued by Sofosbuvir treatment	[103]
		WNV exposure	100-day-old COs	Viral foci within outer cortical plate-like regions enriched in neurons and astrocytes; Increased release of pro-inflammatory cytokines (IL-6, TNF-α, IL-18), chemokines (CXCL10, CCL17, CX3CL1, CCL2), and biomarkers (IL-1RA, sTREM-1, sRAGE, BDNF)	[120]
		JEV exposure	24- and 55-day-old cortical organoids	Reduced proliferation and increased cell death; reduced organoid size; preferential infection of astrocytes and neural progenitors (especially oRGCs); stage-dependent antiviral responses, with interferon signaling activation detected only in more mature (> 8 weeks) organoids	[121]
		Mpox exposure	70- and 110-day-old COs	Observed cell-to-cell viral spread; neurite bead formation preceding neurite-initiated cell death; altered cellular homeostasis and upregulation of neurodegeneration-associated gene expression with limited inflammatory and antiviral responses; reduced viral load following tecovirimat treatment	[122]
Maternal Stress		Dexamethasone exposure (10 nM, 100 nM, and 1,000 nM) for 4 and 12 h	45-day-old COs	GR expressed in NPCs; dexamethasone induced GR-transcription; altered fate genes (HES6, PAX6)	[129]
		Cortisol exposure (1 µM for 72 h + 500 nM for 1 h trigger after 2 days)	300-day-old cortical organoids	Induced phosphorylation of GR and differential expression of stress-, hypoxia-, and ECM-related genes; astrocytic and oligodendrocytic populations were major mediators of GC response	[130]
Exposure to Drugs, Medications, or Environmental Neurotoxins	Alcohol consumption	Chronic ethanol exposure for 20 days (50 mM)	10-day-old COs	Reduced neurite outgrowth; impaired neuronal maturation; altered gene expression (e.g., GSX2, RSPO2); Hippo signaling dysregulation	[136]
		Acute ethanol exposure for 6 h (115 mg/dl)	2-month-old COs	Increased apoptosis; mitochondrial dysfunction; metabolic stress, deregulation of gene clusters involved in proliferation, nervous system development, and neurological disease pathways	[137]
		Ethanol exposure (100 mM) for 7 days	4-week-old cortical organoids	Altered histone modifications and chromatin accessibility; impaired cAMP/calcium signaling; disrupted glutamatergic synapse formation and astrocytic function	[138]
	Nicotine	Nicotine exposure for 5 days	11- and 35-day-old COs	Early exposure: induced cell death; increased the proportion of Tuj1^+^ neurons, thus leading to premature neural differentiation; depletion of key forebrain markers PAX6 and FOXG1. Late exposure: selective depletion of TBR1^+^ layer VI neurons and an increased proportion of CTIP2^+^ layer V neurons, suggesting disrupted cortical layering	[142]
Exposure to Drugs, Medications, or Environmental Neurotoxins (cont.)	Nicotine (cont.)	Chronic nicotine exposure (0.1, 1, or 10 µM) for 14 days	28-day-old COs	Short-term: molecular changes linked to neural identity (cortical marker EMX1 and forebrain marker FOXG1), mood, and anxiety regulation; cortical E/I imbalance. Long-term: persistent E/I imbalance; reduced neural identity marker expression (preplate marker TBR1 and progenitor marker EOMES); altered dopamine receptor expression	[143]
	Cannabis	Prolonged THC exposure (1 µM) for 27 days	3-day-old COs	Reduced neuronal maturation; CB1 receptor downregulation; impaired neurite outgrowth; decreased spontaneous activity	[148]
	Cocaine	Cocaine exposure (3 µM, 1 h every other day) for 12 days	32-day-old neocortical organoid model	CYP3A5-induced ROS increase; reduced progenitor proliferation; premature neuronal differentiation; impaired tissue development; cocaine-induced phenotypes reversed by CYP3A5 knockdown	[154]
		Cocaine exposure (25 µM) for a total of 48 h	36-day-old COs	Changed gene expression through epigenetic remodeling; disrupted mechanisms of neural plasticity; perturbed neurodevelopmental signaling pathways; inflammatory activation of astrocyte populations	[153]
	Opioids	Prolonged methadone exposure (1 to 10 µM)	Before (-9-day), during (0-day), and after (9-day) the EB formation	Suppressed the firing of spontaneous action potentials; lower frequency and amplitude of excitatory postsynaptic currents; weakened synaptic network activity; decreased voltage-dependent Na⁺ current	[170]
		Methadone exposure (1 and 10 µM) for 12 weeks	3-6-month-old cortical organoids	Suppressed neuronal function; impaired ion channel function	[171]
		Methadone exposure (1 µM) for 50 days	9-day-old cortical organoids	Transcriptional changes in synaptic/ECM/cilia pathways; impaired synaptogenesis	[172]
		Methadone exposure (5 µM) for one week	10-month-old COs	Astroglial responses; disrupted cAMP signaling; impaired glutamate regulation; and elevated IL-8, IL-6, GFAP, and NLRP expression; neurotoxicity	[173]
		Chronic buprenorphine exposure (2 ng/ml) for 10 days	50-day-old fused hCS-hSS	Increased markers of interneuron progenitors in hSS; increased interneuron migration into hCS, and elevated network activity; dysregulated NOP signaling	[174]
		Buprenorphine exposure (0.9 nM) for 5 days	30-day-old COs	Increased apoptosis; astrogliogenesis and glial maturation; enhanced dopamine release; BMSCSec elicited effects, except for dopamine release, which was reduced	[175]
		Methadone & buprenorphine exposure (1 and 10 µM) for 24 days	0-day-old cortical organoids	Milder effects of buprenorphine on growth and neuronal activity than methadone	[176]
		Chronic (0.3–74 nM, for 53 or 77 days) and acute (4 to 74 nM, for 4 h) fentanyl exposure	Chronic treatment on day 1 of neuronal induction, and acute treatment on 95-day-old midbrain organoids	Chronic treatment: arrests neuronal subtype specification; altered synaptic activity and neuron projection Acute treatment: enhanced dopamine release; no significant gene-expression changes relevant to cell lineage development	[177]
Exposure to Drugs, Medications, or Environmental Neurotoxins (cont.)	Environmental pollution	Chronic Cd exposure (10 nM, environmentally relevant concentration; 100 nM, occupationally relevant concentration; 1000 nM, high concentration) for 49–77 days	14-day-old COs	Reduced growth; impaired neuronal differentiation and cortical layering; reduced GFAP expression; loss of SOX2⁺ progenitors with more TUJ1⁺ immature neurons; early rise followed by decline in TBR2⁺ IPs and CTIP2⁺ deep-layer neurons; disrupted zinc and copper balance due to metallothionein overproduction; impaired synaptogenesis and postsynaptic protein expression	[164]
		As and/or Pb exposure (As 50 µM, 200 µM; Pb 50 µM, 200 µM; As 50 µM + Pb 50 µM) for 24 h	40-day-old brain organoids with optic vesicles	Structural disorganization and cellular displacement within the VZ; disrupted cell cycle; altered autophagy and Wnt signaling	[165]
		DPM exposure (30 mg DPM dissolved in PBS, 30 µg DPM + 0.5% dimethyl sulfoxide) for 7 days	30-day-old COs	Disrupted oxidative phosphorylation; impaired mitochondrial function; altered cytosine methylation	[166]
		Rotenone exposure (5 µM) for 24 h and 48 h	2, 4, and 8-week-old brain spheres	Heightened ROS and mitochondrial dysfunction during early stages; impaired Ca²⁺ homeostasis, synaptogenesis, and peroxisome proliferator-activated receptor signaling	[167]

### Immune activation/inflammation

Growing evidence from clinical and preclinical studies indicates that maternal/fetal immune activation due to infection or systemic immune responses contributes to the risk of long-term neurodevelopmental disorders and psychiatric diseases in offspring, including autism spectrum disorders (ASD) [56]. Increased levels of proinflammatory cytokines during pregnancy have been associated with alterations in neonatal brain structural and functional connectivity and impaired cognitive function in infants [57, 58]. To model this process in the brain organoid context, a dorsal forebrain organoid model of maternal/fetal immune activation based on constitutive interleukin-6 (IL-6) signaling has been shown to recapitulate key pathological features, including altered RG dynamics, selective overproduction of excitatory neuronal subtypes, disrupted cortical organization, and transcriptomic changes associated with ASD, highlighting human-specific regulatory pathways not captured in animal models [59]. Another study aimed to generate microglia-containing brain organoids to study the impact of early immune challenges on the developing brain. Exposure to a panel of inflammatory triggers revealed that interferon-γ (IFN-γ) induced the release of multiple cytokines and chemokines, including tumor necrosis factor-α (TNF-α), IL-8, IL-10, C-X-C motif chemokine ligand 9 (CXCL9), IL-6, monocyte chemoattractant protein-1 (MCP-1/CCL2), and MCP-3. Transcriptomic profiling revealed that IFN-γ caused significant transcriptional and structural changes, which were modulated by the presence of microglia. In organoids lacking microglia, IFN-γ reduced the proportion of SOX2^+^ neural precursors, which is consistent with its known inhibitory effects on progenitor populations. This reduction, however, was not observed in microglia-containing organoids, supporting a protective role of microglia. In contrast, the number of NEUN^+^ neurons was decreased by IFN-γ irrespective of the presence of microglia, suggesting that mature neurons remain vulnerable to immune insult. Specifically, IFN-γ was shown to dysregulate the expression of ASD-related genes; however, the incorporation of microglia reversed these alterations, suggesting a protective role for microglia against immune-mediated insults. Overall, this study highlights the importance of incorporating microglia into brain organoids to more accurately model responses to inflammatory stimuli [60].

### Maternal fever and thermal shock

Maternal fever during pregnancy can cause thermal stress in both the mother and the fetus, potentially leading to embryonic lethality, structural or functional defects, and growth impairments. Early gestational hyperthermia is associated with abnormal brain development [61–63], including neural tube defects (NTDs) [64, 65], brain atrophy [66], and neuronal ectopia [62]. Despite these associations, the mechanisms underlying hyperthermia-induced neurodevelopmental disruption remain poorly understood, mostly due to the lack of appropriate research models. To address this, Xu et al. developed a periodic heating model based on COs derived from PSCs. They reported that periodic heat exposure decreased the size and impaired neural tube-like structures by reducing the total loop area of brain organoids. Bulk RNA sequencing revealed dysregulation of Wnt signaling and a reduction in the number of G2/M progenitor cells. Further, an increase in neural differentiation and a reduction in progenitor proliferation have been observed, directing organoids toward premature differentiation and altering development trajectories [67]. These findings establish brain organoids as valuable models for elucidating the mechanisms of prenatal thermal stress and its impact on human neurodevelopment.

### Viral infections in utero

Maternal infections with specific viruses and parasites, particularly classical TORCH pathogens, such as *Toxoplasma gondii*, Rubella virus, cytomegalovirus (CMV), and herpes simplex virus (HSV), can be transmitted to the fetus through intrauterine routes, potentially leading to a broad range of neurodevelopmental abnormalities. However, conventional animal models often fail to recapitulate these infections, mainly because many of these pathogens exhibit high species specificity.

#### Zika virus

During the 2015–2016 Zika virus (ZIKV) outbreak in the Americas, the power of organoid models truly came to light. Zika virus infection in pregnant women has led to an epidemic of newborns with microcephaly and other malformations, as the first-trimester human placenta is permissive to viral infection and even supports its replication [68, 69]. Researchers rapidly turned to brain organoids to investigate ZIKV, since rodents failed to reproduce the human-specific neural tropism of the virus and the range of symptoms observed in humans [70]. COs recapitulate the potential mechanism underlying the microcephalic phenotype, with ZIKV preferentially infecting RGs, causing massive apoptosis of these progenitors and a consequent reduction in organoid size [70–73]. This restricted growth was accompanied by disrupted proliferative zones and cortical layering [20, 72, 74]. These findings aligned with observations in the Zika-affected human fetal brain, thereby validating the use of organoids as a relevant model. As ZIKV primarily targets the forebrain, Qian et al. generated region-specific forebrain organoids at different ages to study the preferential infection of SOX2^+^ NPCs, including HOPX^+^ oRGs, by ZIKV. These findings suggest that microcephaly resulting from *in utero* ZIKV infection is linked to the virus’s selective infectivity in specific populations of developing neurons. At later developmental stages, neurons and astrocytes also proved vulnerable to ZIKV infection [20, 75]. Infected organoids recapitulate key characteristics of congenital Zika syndrome, such as thinning of the neuronal layer, disruption of apical surface adherent junctions, and enlargement of the ventricular lumen, suggesting that prenatal ZIKV exposure clearly leads to neurological abnormalities [20]. Mechanistic insights have revealed that ZIKV infection activates Toll-like receptor 3 (TLR3), triggering apoptosis and impairing neurogenesis, thereby reducing organoid size [71]. In parallel, microglial AXL receptor upregulation was detected in infected organoids, suggesting a possible role in viral entry [76]. Other validated mechanisms include disruption of adherens junctions in ventricular RGs, leading to premature differentiation and aberrant neuronal migration [77]. Moreover, viral particles show tropism for aquaporin-1 (AQP1)^+^ cells, which likely represent choroid plexus cells, further broadening the understanding of ZIKV infection mechanisms [72]. Importantly, brain organoids have also served as a platform for therapeutic testing. Several compounds attenuate apoptosis [72, 78], reduce viral replication [79, 80], and protect against infection [72]. In summary, brain organoid studies have revealed the selective vulnerability of neural progenitors to ZIKV infection, identified diverse cellular targets and molecular mechanisms underlying microcephaly, and established organoids as a powerful platform for screening drugs for treating congenital Zika syndrome [72, 78].

#### Cytomegalovirus

Cytomegalovirus (CMV) poses a major threat to the developing fetus, as primary maternal infection during pregnancy carries an estimated 40% risk of vertical transmission [81]. Congenital CMV affects an estimated 0.5–2% of all newborns worldwide and represents a leading cause of neurodevelopmental impairment at birth or later in life, including hearing loss, intellectual disability, microcephaly, or cerebral palsy. Despite its clinical importance, the mechanisms underlying CMV-induced neuropathogenesis remain poorly understood. Research is further constrained by the strict species specificity of CMV, which limits studies to in vitro human cell culture systems and precludes the use of conventional animal models [82]. Human brain organoid studies have recapitulated the clinical observations, showing impaired growth following CMV infection, which is consistent with microcephaly. Sun et al. identified PDGFRα and EGFR as entry routes for CMV and observed partial colocalization of TBR2^+^ IPs with the CMV immediate-early protein IE1[82]. Recently, Brown et al. reported profound morphological abnormalities in CMV-infected organoids, including necrotic regions, cyst formation, and reduced cellularity. Disorganization of the Nestin^+^ and Tuj1^+^ domains indicates that primary damage to NPCs disrupts subsequent neurogenesis [83]. Complementary experiments with brain organoids revealed progressive transcriptional changes: lipid metabolism pathway genes were upregulated on day three, developmental program genes were upregulated on day five, and inflammatory response genes were upregulated on day seven postinfection. Interestingly, PAX6^+^ cells remained largely uninfected, whereas infected populations were located adjacent to neural rosettes and expressed doublecortin, a marker of newborn neurons [84]. Taken together, the results of organoid studies revealed that CMV primarily targets neural stem/progenitor cells, resulting in impaired neuronal and glial differentiation, disorganized cortical architecture, and growth deficits, thereby faithfully modeling the clinical features of congenital CMV infection.

#### Herpes simplex virus

Herpes simplex virus (HSV)-1 and HSV-2 are among the most common TORCH infections during pregnancy and are the primary causes of neonatal herpes encephalitis, a severe condition with an estimated 60% mortality in the absence of antiviral therapy. Although Acyclovir therapy markedly reduces mortality, achieving favorable clinical outcomes remains challenging, even with early diagnosis and intervention [85]. Long-term neurological sequelae, including intellectual disability, cerebral palsy, autism, cortical blindness, and epilepsy, are reported in 59–69% of survivors of neonatal encephalitis [86, 87]. Intrauterine infection is relatively rare, but it has the most severe neurodevelopmental and cognitive consequences [88, 89]. The mechanisms underlying latency and reactivation are not yet fully understood, as the human-specific nature of the infection constrains experimental modeling and has produced inconsistent results in animal studies [90]. Recent studies have employed brain organoids to investigate HSV neuropathogenesis (summarized in Table 1), revealing that neurite degeneration, cell‒cell fusion, impaired neurogenesis, and microglial activation are accompanied by elevated levels of inflammatory mediators [90–92]. Single-cell and functional analyses further revealed the highest viral load in NPCs, reduced neuronal activity, persistent TNF signaling, and upregulation of stress- and infection-associated genes in both RGs and IPs after infection. IPs also downregulated cell division pathways. HSV-1 infection also induced a global elongation of poly(A) tails and preferential selection of distal 3′ adenylation sites, which is consistent with viral manipulation of the translational machinery to increase viral replication at the expense of host mRNA stability and protein synthesis [93, 94]. These findings underscore both the complexity of HSV-induced neuropathogenesis and the value of brain organoids for modeling host‒virus interactions and identifying synergistic therapeutic strategies.

#### Coronavirus SARS-CoV-2

Clinical data from adult patients with SARS-CoV-2 infection indicate potential neurological manifestations, raising concerns about possible impacts on fetal brain development following maternal infection *in utero* [95]. Indeed, evidence of *in utero* transmission of the novel coronavirus through the placenta remains conflicting [68, 96–98]. Nonetheless, the two primary mediators of SARS-CoV-2 cell entry, angiotensin-converting enzyme 2 (ACE2) and transmembrane serine protease 2 (TMPRSS2), are expressed in placental syncytiotrophoblasts during the first trimester and in extravillous trophoblasts during the second trimester, thereby permitting viral entry [97, 98]. Bulk expression of both receptors decreases as pregnancy progresses, suggesting particular fetal vulnerability during early to midgestation [99]. In support of this notion, SARS-CoV-2 has been detected in the umbilical cord at midgestation [100], has been demonstrated to replicate within the placenta [101], and to infect the choroid plexus (ChP) in organoid models [102], providing a potential route for viral entry into the fetal brain.

Studies on brain organoids have demonstrated that SARS-CoV-2 can infect NSCs [103–105], neurons [102–106], and even astrocytes [102, 103, 106]. Reports suggest viral tropism toward NSCs, with colocalization of viral proteins with SOX2^+^ neural rosettes [107] as well as Nestin^+^ progenitors [103, 105]. However, some reports do not support this observation [106, 108], and discrepancies may be due to differences in organoid protocols, developmental stages, infection durations, or viral doses, underscoring the need for replication across independent studies. Additionally, coronavirus infection induced shifts in the cell type composition within organoids, characterized by an increase in more mature neurons and a reduction in transient progenitor cells, suggesting premature differentiation [104]. Together, these observations highlight that SARS-CoV-2 infection during pregnancy may pose a significant hazard to the fetal brain, particularly during peak neurogenesis, due to placental permissiveness and subsequent ChP-mediated viral entry into neural tissue. The identified neuronal loss, metabolic dysregulation, and premature differentiation in brain organoids raise concerns about potential long-term neurodevelopmental consequences in children exposed to maternal infection. These risks may be further compounded by maternal immune activation, which has been consistently linked to increased susceptibility to neurodevelopmental disorders in the fetus [10].

#### Other viruses

In addition to the well-characterized congenital neurotropic viruses, several other pathogens have been sporadically associated with perinatal brain injury, including West Nile virus (WNV) [109–111] and Japanese encephalitis virus (JEV) [112–115]. Although not traditionally recognized as a congenital neurotropic pathogen, vertical transmission of Monkeypox virus (Mpox) has been documented in humans, with viral DNA detected in placental and fetal tissues. The reported cases include miscarriage, stillbirth, hydrops fetalis, and severe neonatal infection, suggesting that maternal infection can compromise fetal viability. However, Mpox has not been linked to a consistent pattern of cortical malformations or congenital brain anomalies. Whether adverse outcomes are driven by direct neural infection, placental pathology, or systemic maternal illness remains unresolved [116–118].

Infected brain organoids with WNV presented prominent viral foci within cortical plate-like regions enriched in neurons and astrocytes, accompanied by increased expression of proinflammatory cytokines and chemokines. These models were further utilized to evaluate the cytotoxicity and antiviral efficacy of several candidate compounds [119, 120]. Similarly, JEV has been shown to preferentially infect astrocytes and neural progenitor populations, particularly oRGCs, in infected brain organoids, and also elicits stage-dependent antiviral responses [121]. In addition, Mpox-infected brain organoids demonstrated viral dissemination through direct cell‒to‒cell transmission, resulting in the disruption of cellular homeostasis and upregulation of transcripts associated with neurodegenerative pathways [122]. Owing to the scarcity of systematic human data and the limitations of conventional animal models, recent studies have employed human brain organoids to explore the impact of these less-studied viruses, thereby offering new opportunities to elucidate their neuropathological mechanisms and identify potential therapeutic strategies.

### Maternal stress

Maternal stress has been consistently associated with a wide range of neurodevelopmental abnormalities that can persist into adulthood [123]. Nonetheless, the presence of multiple confounding variables and the absence of reliable biomarkers for stress have limited the ability to infer causality [124]. Cortisol, the principal stress hormone in humans, belongs to the glucocorticoid (GC) family, a group of lipid-soluble molecules that readily cross the placental barrier and the BBB [123]. Under normal conditions, 50–90% of maternal cortisol is inactivated by placental 11β-hydroxysteroid dehydrogenase type 2 (11βHSD2) [125]. However, chronic stress, inflammation, or maternal alcohol consumption can reduce 11βHSD2 activity, increasing the likelihood of cortisol reaching the fetal brain [126]. In addition to endogenous cortisol, synthetic GCs such as dexamethasone and betamethasone are prescribed during pregnancy to prevent congenital adrenal hyperplasia at midgestation [127]. Since placental deactivation of synthetic GCs is less efficient, their impact on the fetal brain may be more pronounced. Both natural and synthetic GCs act through glucocorticoid receptors (GRs) and mineralocorticoid receptors [128]. Recent organoid-based work has provided new insight into these mechanisms. A study reported the expression of the GR-encoding gene NR3C1 from days 17–158 of human cerebral organoid development, which was particularly enriched in NPCs, with GR localized in the nuclei of neural rosette cells. Functionally, acute dexamethasone exposure induced GR-dependent transcriptional responses. Single-cell transcriptomics have revealed differential expression of genes involved in fate determination during brain development, such as HES6 and PAX6. Gene ontology analysis further revealed enrichment of pathways related to cell differentiation, neurogenesis, and nervous system development in NPCs and neurons. These findings suggest that GC exposure can interfere with neuronal differentiation during early cortical development [129]. Furthermore, Purmann et al. established a “stress-in-a-dish” (SIAD) model in which cortical organoids were used to investigate the molecular signatures of stress exposure, which resulted in robust GR activation and cell type-specific transcriptional responses, particularly in astrocytic populations [130]. Single-cell analyses revealed the induction of hypoxia-related and extracellular matrix–associated gene programs, indicating that glial cells are key mediators of cortisol-driven stress signaling and linking prenatal stress exposure to molecular pathways associated with neurodevelopmental vulnerability and stress-related psychopathology.

Furthermore, GCs affect the placenta by reducing glucose transport to the fetal compartment, resulting in intrauterine growth restriction and increased expression of hypoxia-related genes, as shown in mouse models at E12.5 [131]. Therefore, the neurodevelopmental consequences of maternal stress may arise not only from direct effects of GCs on the brain but also from secondary outcomes of placental dysfunction, nutrient deficits, and hypoxia.

### Exposure to drugs, medications, or environmental neurotoxins

Each year, more than one million fetuses are exposed to psychoactive substances *in utero* [132]. Exposure to licit and illicit drugs such as nicotine, alcohol, marijuana, cocaine, heroin, hallucinogens, inhalants, and misused prescription drugs has been associated with fetal growth restriction, stillbirth, preterm birth, and increased neonatal morbidity [133]. However, elucidating the molecular and cellular mechanisms that underlie these outcomes remains challenging. Studies on prenatal drug exposure are often complicated by concurrent multidrug use, socioeconomic factors, and a limited number of participants. For these reasons, most assessments are conducted retrospectively in adolescents or adults, or modeled in animals and 2D cell cultures, which cannot fully capture the complex developmental landscape of the human fetal brain. Over the past few years, several studies have demonstrated the utility of human brain organoids as powerful models for dissecting the impact of both illicit and nonillicit drugs and environmental neurotoxins on early brain development [134].

#### Maternal alcohol consumption

Maternal alcohol consumption has long been recognized as a major risk factor for long-term fetal neurodevelopment deficits, collectively termed fetal alcohol spectrum disorders (FASD), which have a global prevalence of 0.15% [135]. Symptom severity ranges from subtle neurobehavioral abnormalities to structural alterations such as microcephaly and eventually fetal alcohol syndrome, which is defined by a combination of physical and neurobehavioral deficits resulting from continuous alcohol consumption throughout pregnancy [133]. Ethanol, as a small molecule, can easily cross biological membranes and can therefore reach the fetal brain. Studies on brain organoids have shown that chronic ethanol exposure causes widespread apoptosis, depletion of SOX2⁺ neural progenitors, and increased Tuj1⁺ neurons, which is consistent with premature neuronal differentiation, alongside deregulation of cell adhesion genes and reduced neurite outgrowth in organoid cultures [136]. Acute exposure similarly induces apoptosis, mitochondrial ultrastructural abnormalities, metabolic dysfunction, and glycogen accumulation, with transcriptomic changes affecting proliferation, neurodevelopment, and neurological disease pathways [137]. In addition, ethanol has been shown to alter posttranslational histone modifications and chromatin accessibility, ultimately impairing calcium signaling, synaptic maturation, and astrocytic function [138], collectively highlighting how prenatal alcohol exposure disrupts multiple converging mechanisms of early human brain development.

#### Maternal nicotine exposure

Prenatal nicotine exposure has also been associated with persistent effects in the developing fetal brain [139]. Globally, the prevalence of smoking during pregnancy is estimated at 1.7%, with regional variation: 8.1% in Europe, 5.9% in the Americas, 1.2% in both Southeast Asia and the Western Pacific, 0.9% in the Eastern Mediterranean, and 0.8% in Africa [140]. Nicotine readily crosses the placenta and enters the fetal circulation, directly exposing the developing brain [141]. Brain organoid studies have shown that early exposure induces cell death and premature neuronal differentiation with loss of forebrain identity, whereas later exposure disrupts cortical layering by selectively affecting deep-layer neuronal populations [142]. Further studies revealed that chronic nicotine exposure in COs deregulates the expression of markers linked to neural identity and glutamatergic, GABAergic and dopaminergic markers involved in mood and anxiety regulation and ultimately disrupts the cortical excitatory/inhibitory (E/I) balance. In the long term, nicotine exposure sustained disruptions in the E/I balance, reduced the expression of neural identity markers, and altered dopamine receptor expression. These observations provide insight into how nicotine exposure may shape neural circuitry and lead to mood and anxiety disorders later in life [143].

#### Cannabis abuse

Owing to the widespread perception of cannabis as relatively safe during pregnancy, an estimated 1–5% of pregnant women report its use, with the prevalence reaching 16.5% among pregnant adolescents aged 15–17 years [144]. However, given that its principal psychoactive component, Δ-9-tetrahydrocannabinol (THC), can cross the placenta and enter the fetal circulation [145]. The potential for subtle yet long-term neurodevelopmental effects should not be underestimated, as alterations in executive and visuoperceptual functions have been reported in adolescents born to mothers who consumed cannabis during pregnancy [146]. Recent studies using CO models reported that THC exposure led to the upregulation of PAX6, accompanied by the downregulation of the neuronal markers CTIP2 and Tuj1. Moreover, the expression of the cannabinoid receptor CB1, a regulator of the balance between the proliferation, differentiation, migration, and maturation of neural cell populations [147], was reduced in THC-treated organoids. Accordingly, brain organoids exposed to THC exhibit reduced neuronal maturation, impaired neurite outgrowth, disrupted excitatory activity, and decreased spontaneous firing [148].

#### Cocaine abuse

Cocaine usage represents a significant public health concern, as it is recognized as a teratogen for the developing fetus [133]. It can cross the human placenta [149, 150], and owing to its high lipophilicity, it crosses the BBB and accumulates in the brain [151]. Since the developmental timeline of neurogenesis and maturation of neuronal receptors and neurotransmitters differs between humans and rodents and cocaine responses vary widely across animal models (most notably, owing to the species-specific expression of cytochrome P450 (CYP450) enzymes, which mediate the initial metabolism of cocaine in neural cells), human-derived brain organoids have emerged as a powerful model system for investigating the mechanisms of prenatal cocaine exposure [152, 153]. A neocortical organoid model revealed that periodic cocaine exposure during the proliferative phase of organoid development led to ROS accumulation, inhibition of NPC proliferation, and premature differentiation, ultimately interrupting neural tissue development. Notably, knockdown of CYP3A5 reversed these pathological phenotypes, directly implicating this enzyme in cocaine-induced neurodevelopmental abnormalities [154]. Recent studies using advanced single-cell transcriptomic and epigenomic approaches in COs revealed that cocaine exposure partially alters gene expression via epigenetic remodeling, disrupting neural plasticity. Cocaine also perturbs neurodevelopmental signaling and triggers inflammatory responses through astrocytic activation. Together, these changes suggest that prenatal cocaine exposure profoundly impairs brain maturation [153]. Additionally, maternal cocaine use causes severe vasoconstriction, reducing placental blood flow and fetal oxygenation, which induces hypoxic stress in the developing brain [151].

#### Prescribed medications

Opioids, which are widely prescribed for pain relief, also have high potential for misuse because of their euphoric effects and ability to generate strong positive reinforcement [155]. With rising rates of prescription and use over the past two decades [156], pregnant women represent a particularly vulnerable group because of the ability of exogenous opioids to readily cross the placenta and accumulate in fetal tissues, raising significant concerns for neurodevelopment [157]. Prenatal opioid exposure (POE) has been associated with neurocognitive deficits and neuropsychiatric comorbidities in both humans and animals [158, 159], with preclinical studies linking these outcomes to widespread disruptions in neuronal and glial proliferation, differentiation, and maturation [133, 160]. However, differences across species in behavior, neurodevelopmental stages, cellular heterogeneity, opioid receptor expression, and opioid pharmacokinetics and bioavailability [133] limit the direct translation of findings from animal studies, making human brain organoids a strong model for POE etiology and progression. Organoid-based studies have focused primarily on clinically relevant opioids such as methadone and buprenorphine, as well as potent synthetic opioids such as fentanyl, revealing drug- and exposure-dependent alterations in cortical growth, neuronal excitability, synaptogenesis, glial responses, and neurotransmitter signaling. Together, these studies, summarized in Table 1, highlight convergent vulnerabilities of developing neurons and glial cells to opioid exposure, demonstrating common mechanisms in which neurons exhibit impaired neurogenesis, maturation, and migration, whereas astrocytes and other glial cells display inflammatory responses [134].

#### Environmental neurotoxins

Accumulating evidence indicates that prenatal exposure to environmental pollutants contributes to adverse neurodevelopmental outcomes [161, 162]. Brain organoids have emerged as powerful systems for investigating how environmental toxins may exert long-lasting effects on fetal brain development [163]. For example, Yan et al. examined chronic Cd exposure in mature COs for 49 or 77 days. Prolonged treatment with this heavy metal inhibited organoid growth and disrupted neural differentiation and cortical layering. These effects also included impaired GFAP expression, reduced numbers of SOX2^+^ neural progenitors, increased numbers of TUJ1^+^ immature neurons, and dynamic changes in TBR2^+^ IPs and CTIP2^+^ deep-layer cortical neurons, with initial increases followed by decreases. Transcriptomic analyses further revealed that Cd altered zinc and copper homeostasis via increased metallothionein synthesis and impaired synaptogenesis, as reflected by reduced postsynaptic protein expression [164]. Other pollutants have similar detrimental effects. Short-term exposure (24 h) to arsenic (As) or lead (Pb) caused structural disorganization and cellular displacement within the VZ of the brain organoids. Proteomic analyses have indicated that these metals interfere with cell proliferation and differentiation, disrupt the cell cycle, and alter autophagy and Wnt signaling, potentially increasing susceptibility to neurodevelopmental disorders and carcinogenesis [165]. Diesel particulate matter (DPM), a key component of diesel exhaust emissions, has also been shown to disrupt oxidative phosphorylation and impair mitochondrial function in brain organoids, linking *in utero* exposure to ASD-related pathophysiology [166]. Similarly, rotenone, a widely used pesticide, demonstrates stage-dependent toxicity after exposure to brain organoids. Early exposure heightened reactive oxygen species and mitochondrial dysfunction during the initial phases of organoid differentiation while also impairing Ca²⁺ reabsorption, synaptogenesis, and peroxisome proliferator-activated receptor signaling. At noncytotoxic concentrations, rotenone selectively damages dopaminergic neurons, whereas at higher concentrations, it causes widespread astrocytic and neuronal toxicity [167]. Together, these findings highlight the vulnerability of the developing brain to diverse environmental neurotoxins and emphasize the importance of preventive measures to reduce maternal exposure during pregnancy.

### Multi-insult combinatorial injury modeling

**Fig. 2:**
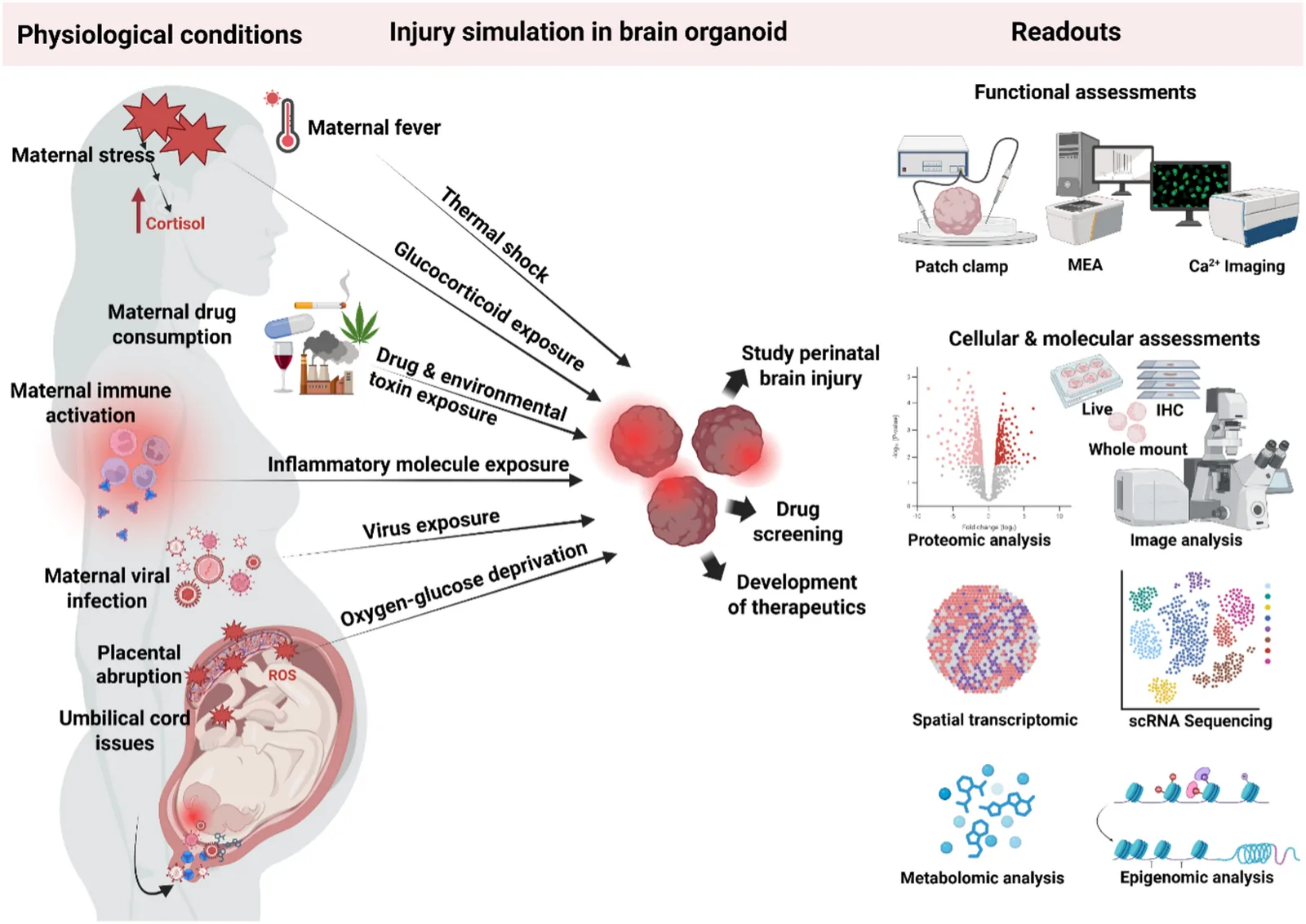
Modeling perinatal brain injury using cerebral organoids. Maternal physiological conditions such as placental abruption, umbilical cord issues, immune activation, maternal fever, viral infections, maternal stress, drug consumption and environmental toxin exposure can lead to perinatal brain injury. These insults can be simulated in brain organoids via oxygen‒glucose deprivation, proinflammatory molecule exposure, thermal shock, viral infection, glucocorticoid treatment, and drug and environmental toxin exposure. Organoids allow mechanistic studies of injury and therapeutic testing through a broad range of readouts, including functional assessments and cellular/molecular profiling. ROS: reactive oxygen species. MEA: multielectrode array, IHC: immunohistochemistry. Created in https://BioRender.com

Perinatal brain injury often arises from complex, multifactorial insults [168]. Brain organoids can be exposed to combined insults, such as hypoxia with glucose deprivation or viral infection alongside inflammatory stimuli, to better simulate the pathophysiology observed in vivo [169]. These combinatorial approaches allow the study of converging molecular mechanisms and the evaluation of multitarget interventions that may be more effective than single-agent strategies. Early attempts in this direction are still rare, but conceptually, organoids constitute one of the few models in which researchers can ethically explore “two-hit” hypotheses for PBI, underscoring the future opportunity for research on combined-insult organoid models (Fig. 2).

## Development and screening of drugs and therapeutics

The fetus exhibits higher vulnerability to maternal exposure to substances that may appear less harmful in adults but can impair fetal brain development. Thus, drug development in pregnant women has long been hindered by ethical and safety concerns regarding fetal exposure, creating a significant barrier to research and therapeutic innovation. A critical bottleneck, termed the “valley of death”, exists between preclinical research and clinical translation, where many promising compounds fail to advance [48, 178]. The high rate of clinical trial failure is largely due to the limited predictive validity of traditional preclinical models. Organoid technology has the potential to improve trial success by offering patient specific, disease-relevant tissue that enables a deeper understanding of drug responses. Patient-derived organoids could also be used alongside clinical trials to evaluate therapeutic efficacy and adapt treatment strategies in real time. This personalized medicine approach, which integrates genetic and physiological variability, has the potential to improve outcomes while reducing both time and cost [179]. Organoids are valuable tools in different steps of drug development, from identifying therapeutic targets by understanding disease mechanisms to evaluating drug toxicological profiles and testing compound efficacy, and are broadly applied in the screening of a range of pharmaceuticals [180]. This is also in line with recent regulatory initiatives, including the United States of America’s Food and Drug Administration (FDA) Modernization Act 2.0, which recognizes nonanimal technologies as valid tools for drug testing, and the European Medicines Agency (EMA) framework for the regulatory acceptance of new approach methodologies (NAMs) aimed at reducing animal use in testing [181].

### Brain organoids used for the development of therapies

Xu et al. performed an extensive screening of approximately 6,000 compounds for activity against ZIKV infection using both monolayer cultures of NPCs and brain organoids [180]. As ZIKV infection increases caspase-3 activity in NPCs, drug efficacy was assessed based on the ability of ZIKV to inhibit caspase-3 activity and suppress viral replication. They reported that emricasan, a pancaspase inhibitor, effectively reduced caspase-3 activity in both 2D and 3D cultures but did not inhibit ZIKV replication. In addition, niclosamide, a category B anthelmintic drug approved by the FDA, along with 10 structurally distinct cyclin-dependent kinase inhibitors, was shown to suppress ZIKV replication. Thus, they recommended a combined treatment using compounds for both the inhibition of ZIKV replication and caspase-3 activity [180]. Similarly, brain organoids have been used to test therapeutic responses to other viral pathogens. Mesci et al. demonstrated that Sofosbuvir inhibited SARS-CoV-2 replication, reduced cell death, and restored excitatory synapses after SARS-CoV-2 infection in COs [182]. In COs infected with HSV-1, the antiviral drug Acyclovir blocks viral replication, and when combined with anti-inflammatory agents (NEC-1 and CDDO-Me), it alleviates HSV-1-induced damage to cells and tissues [183]. Additionally, Mokry et al. reported that nitric oxide could limit human CMV spread in COs, although it also caused structural alterations in neural rosettes and disrupted tissue organization as side effects [184]. Wang et al. investigated the effects of the neuroprotective compounds edaravone, butylphthalide, P7C3-A20, and ZL006 on COs exposed to OGD. These findings demonstrated that all four agents reduced LDH release and caspase 3 activity, thereby protecting against OGD injury [43]. In a similar study, treatment with GSK2606414 suppressed necrosis, apoptosis, and inflammatory responses while promoting cell proliferation, ultimately attenuating the benzophenone metabolite 4-hydroxybenzophenone (4HBP)-associated neurotoxic effects in COs [185]. Brain organoids also provide a valuable system for assessing the off-target effects of brain-directed therapies, as illustrated by the caspase 3-dependent toxicity observed following combined treatment with erlotinib, an epidermal growth factor receptor (EGFR) inhibitor, and tariquidar, a P-glycoprotein (P-gp) efflux pump inhibitor [186]. In addition, brain organoids offer a platform for evaluating the therapeutic potential of stem cell-based interventions and their derivatives, including extracellular vesicles (EVs) [54, 187]. Stem cell-based therapeutics have demonstrated significant neuroprotective and regenerative benefits in animal models of PBI in several studies [188–192], and organoid systems now offer a critical preclinical bridge toward translation. Brain organoids offer a human-specific model to evaluate the survival, differentiation, and integration of transplanted cells, as well as the neuroprotective, trophic, and immunomodulatory effects of cells and EVs. By providing a controlled, reproducible environment for assessing safety and efficacy, organoids may accelerate the development of personalized regenerative therapies for PBI.

While most early investigations on brain organoids were carried out in academic settings, there is now growing interest from the pharmaceutical and biotechnology sectors, fostering industry-academia collaborations to model neurodevelopmental disorders [74, 193]. Nevertheless, brain organoid technology is still in its early stages, and its use in industry is limited because of challenges ahead for its large-scale implementation [194].

### Challenges in the use of brain organoids for the development of treatments and drug discovery for PBI

Although brain organoids represent a valuable platform for drug discovery, several challenges remain before they can be fully integrated into high-throughput and clinically relevant applications. Some of these challenges include methodological heterogeneity, the limited scalability of the organoids, and issues with reproducibility, which compromise the quality and consistency of the results. To overcome these obstacles, it is crucial to establish standardized protocols and robust quality control measures for brain organoid generation and characterization. Sample heterogeneity in brain organoids primarily arises from variability in iPSC lines due to both environmental and genetic factors and from batch-to-batch variation in organoids caused by diverse reagents, culture conditions, and the absence of standardized protocols for organoid generation across experiments, which prevents the generation of conclusive and generalizable results [195]. Optimizing parameters, including maturation timing, patterning strategy, initial cell numbers, and the use of a more committed cell line, can potentially reduce the variation among batches [196, 197]. While this heterogeneity challenges reproducibility and large-scale applications such as drug discovery and screening, it also reflects the inherent diversity of human brain development and the variability observed across individuals [179]. Thus, brain organoid heterogeneity remains a double-edged sword, simultaneously enhancing physiological relevance while limiting experimental consistency. The scalability of brain organoid technology remains a major challenge, particularly in the context of drug screening, as it is often incompatible with standard high-throughput formats owing to its size and nutrient diffusion limits [198, 199]. Although bioreactors and emerging microfluidic, millifluidic, and automated systems have improved culture capacity, widespread automation of brain organoid workflows is still limited [194]. Nonetheless, recent progress in robotic handling, quantitative imaging, and automated phenotyping has demonstrated that scalable and reproducible brain organoid-based screening platforms are increasingly feasible, supporting their future integration into drug discovery pipelines [194]. For example, Renner et al. established a fully automated HTS workflow for the generation and analysis of midbrain organoids, employing a liquid-handling robot to perform seeding, maintenance, fixation, whole-mount staining, and clearing in a scalable manner using standard 96-well plates. The generated organoids displayed consistent sizes, morphologies, and cellular compositions both within and across batches [200].

## Recent innovations

### Complexity of brain organoids

Brain organoids produced with the help of conventional protocols typically lack certain cell types present in the human brain, including mature oligodendrocytes, microglia, and vascular endothelial cells, all of which are critical for brain development and disease processes [12]. To address this issue, researchers have begun developing more complex organoids that incorporate a broader range of cell types to more accurately recapitulate human brain function.

Oligodendrocytes play crucial roles in brain development, including by myelinating and electrically insulating neuronal axons to facilitate impulse conduction and offering trophic and metabolic support to neurons [201]. As gliogenesis occurs after neurogenesis, mature oligodendrocytes are often absent in brain organoids generated via conventional protocols [12]. Different protocols have been proposed to enrich oligodendrocytes in developing brain organoids. Some studies have promoted oligodendrocyte differentiation in brain organoids by administering a cocktail of factors involved in the survival and proliferation of OPCs (e.g., platelet-derived growth factor and insulin-like growth factor-1) and oligodendrocyte maturation (e.g., triiodothyronine) [16–19]. These organoids contained mature oligodendrocytes located in the vicinity of neurons and astrocytes, forming myelin sheaths around neuronal processes. Interestingly, other studies have reported the emergence of oligodendrocytes in midbrain organoids even in the absence of additional OPC-inducing factors. For example, Monzel et al. generated midbrain organoids from PSC-derived neuroepithelial stem cells, which developed mature oligodendrocytes that formed myelin sheaths enwrapping TUJ1⁺ neurites, with gaps resembling nodes of Ranvier, structures that allow for saltatory signal transmission along axons [202]. The inclusion of the oligodendrocyte lineage is critical for modeling perinatal brain injury, as oligodendrocyte progenitors and premyelinating oligodendrocytes are among the populations most vulnerable to injuries such as hypoxic-ischemic and inflammatory insults in the developing fetal brain [203].

**Fig. 3:**
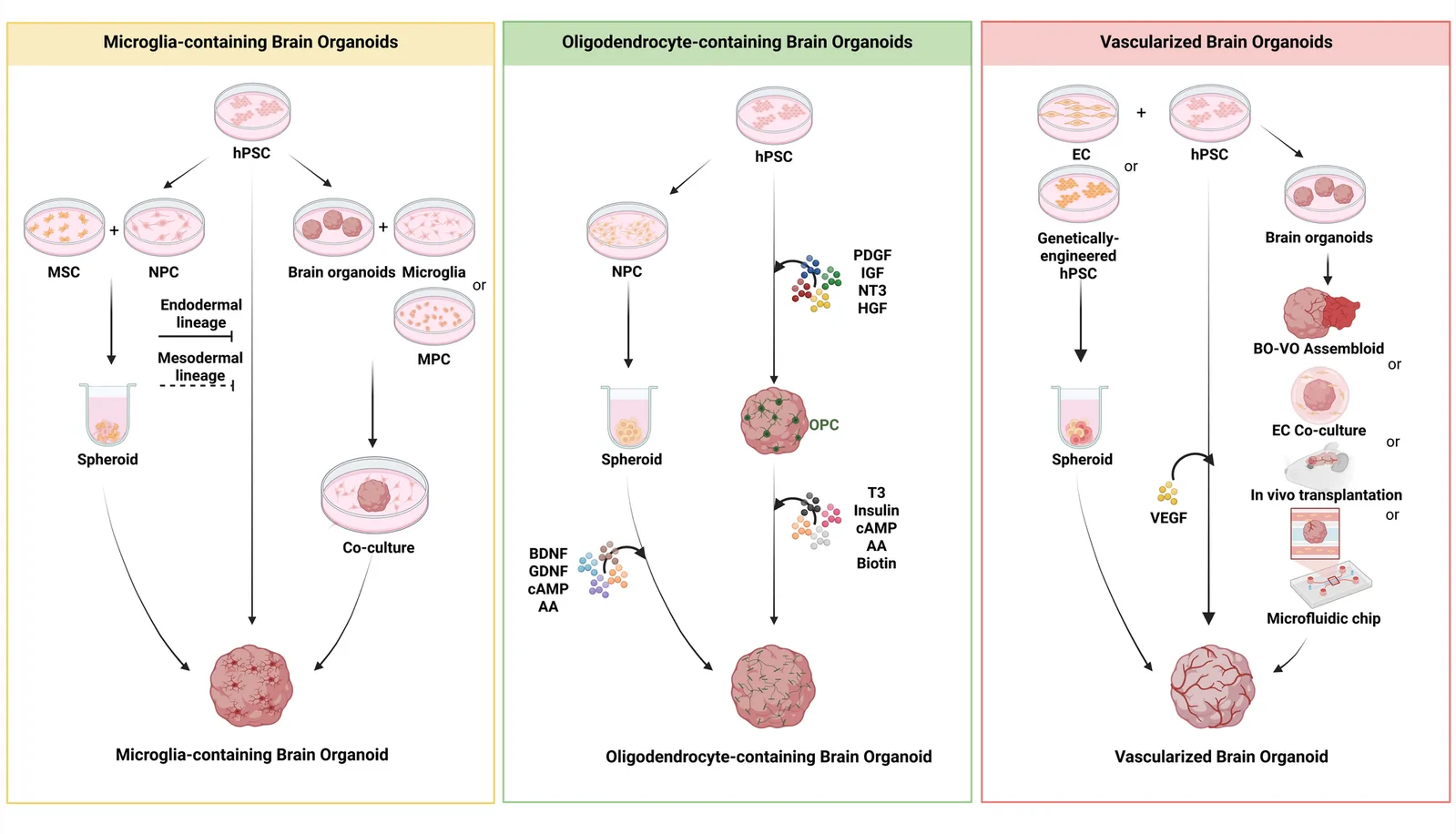
Strategies for generating microglia-and oligodendrocyte-containing and vascularized cerebral organoids. EC: endothelial cell; hPSC: human pluripotent stem cell; MSC: mesenchymal stromal cell; MPC: microglia/macrophage progenitor cell; NPC: neuroepithelial progenitor cell; OPC: oligodendrocyte progenitor cell; PDGF: platelet-derived growth factor; IGF: insulin-like growth factor; NT3: neurotrophin-3; HGF: hepatocyte growth factor; BDNF: brain-derived neurotrophic factor; GDNF: glial cell line-derived neurotrophic factor; cAMP: cyclic adenosine monophosphate; AA: ascorbic acid; VEGF: vascular endothelial growth factor; BO: brain organoid; VO: vascular organoid. Created in https://BioRender.com

Microglia, the resident innate immune cells of the central nervous system, play key roles in astrocyte activation, neuronal development, synaptogenesis, clearance of aggregated proteins, and maintenance of ECM organization and are implicated in the progression of numerous neurological disorders [204]. Thus, microglia are crucial for creating a functional and physiologically relevant brain model. Since they originate from the mesoderm and migrate from the yolk sac during development, microglia are absent in patterned brain organoids derived from the neuroectodermal lineage, where mesoderm and endoderm formation is suppressed [204]. Consequently, multiple studies have sought to integrate microglia into brain organoids (Fig. 3). Xu et al. generated microglia-containing brain organoids, mimicking early embryonic neurodevelopment, by coculturing NPCs and PSC-derived primitive macrophage progenitors, forming organoids in which NPCs were differentiated into functional neurons and astrocytes, while the integrated microglia exhibited phagocytic activity and contributed to synaptic pruning [76]. Brain organoids and microglia can also be developed separately and then cocultured. Several studies have cocultured COs with immortalized human microglia [205], hPSC-derived microglia [206], and hPSC-derived microglia/macrophage precursors [60, 207], which have been shown to be fully functional within the organoid. While coculturing brain organoids with microglia provides a more physiologically relevant brain environment, determining the optimal timing and conditions for microglial integration to accurately recapitulate brain development remains challenging. Interestingly, several other studies have sought protocols to develop endogenous microglia within brain organoids. Ormel et al. facilitated the development of innate microglia by adjusting the concentration of neuroectodermal stimulants, enabling mesoderm-derived progenitor cells to differentiate into microglia within cerebral organoids [208].

One of the key limitations of brain organoids is the absence of vasculature, which restricts their growth and functional maturation at later developmental stages. During embryogenesis, early neural tissue develops without blood vessels and relies instead on nutrient diffusion to support neural progenitor cell maturation. However, as cell layers expand and metabolic demands increase, diffusion alone becomes insufficient, and brain development becomes dependent on a vascular network formed through angiogenesis to deliver oxygen and nutrients [14, 209]. To address this limitation, several strategies have been developed to promote vascularization in brain organoids (Fig. 3). One approach involves coculturing COs with vascular or mesodermal spheroids, enabling perivascular cells to migrate into the CO and form vessel-like networks [209–211]. Another strategy involves mixing endothelial cells (ECs) with PSCs (ESCs or iPSCs) prior to initiating the CO protocol, resulting in spontaneous vessel-like structure formation [212, 213]. Unguided CO protocols can also promote perivascular cell differentiation, which in turn generates vessel-like structures [214, 215]. Supplementing the culture medium with proangiogenic vascular endothelial growth factor (VEGF) further promotes EC differentiation from stem cells within EBs, thereby driving the formation of vascular-like structures in COs [216]. Alternatively, overexpression of the proangiogenic transcription factor ETS variant transcription factor 2 (ETV2) in PSCs before initiation of the CO protocol has been shown to enhance endothelial differentiation and vascularization [217]. The transplantation of COs into the brains of mice or rats provides another avenue through which the host vasculature invades the graft and establishes vessel-like structures [212, 218, 219]. In addition, embedding ECs within a liquid extracellular matrix gel (such as Matrigel) around COs, followed by polymerization, enables the cells to migrate inward and organize into vascular networks [220, 221]. Finally, microfluidic chip systems have been applied to induce VEGF-driven angiogenesis within brain organoids [222, 223]. Together, these approaches represent recent progress toward generating vascularized brain organoids, supporting long-term growth and maturation. Notably, these vessel-like structures neither contain blood-like flow nor form a functional BBB, highlighting important limitations that remain to be addressed [14]. Shear stress, generated by tangential blood flow across endothelial cells, is known to promote BBB-specific phenotypes and support barrier impermeability [224]. Although some vascularized COs (vCOs) demonstrate partial perfusability [217, 225], innate laminar flow—and therefore shear stress—remains absent. Future integration of vCOs into microfluidic chip systems [222] could introduce physiological flow, thereby enhancing BBB maturation and improving the model’s overall functionality.

### Assembloids

A critical advancement has been the development of assembloids, composite 3D cultures made by fusing two or more organoids of different types to model interactions that a single organoid cannot achieve alone [226]. Several assembloid configurations have direct relevance for perinatal brain injury:

Paşca and colleagues pioneered the fusion of a dorsal forebrain organoid (cortical) with a ventral forebrain organoid (subpallial) to create a cortico-subpallial assembloid [23, 227], in which GABAergic interneurons born in the ventral organoid migrate into the cortical organoid and integrate into circuits, similar to how interneurons migrate in the fetal brain from ganglionic eminences to the cortex. This model allows the study of neuronal migration and connectivity, processes that can be disrupted by perinatal insults such as hypoxia or inflammation [228]. Assembloids can also connect the cortex with other regions: thalamocortical assembloids (fusing cortical and thalamic organoids) recreate the reciprocal connections important for sensory processing [229], and cortico-striatal assembloids (cortex with striatum) model motor circuits [230]. In the context of perinatal injury, corticostriatal assembloids could be used to examine how early injury affects later-developing motor pathways relevant to cerebral palsy models. Researchers have even combined multiple regions in a ring or loop assembly, for example, those that connect somatosensory, spinal, thalamic, and cortical organoids to simulate a larger network [231]. These complex assembloids start to approximate neural circuits rather than isolated regions, opening avenues to investigate network-level phenomena following perinatal insults [232]. Interestingly, a myelination assembloid model was recently developed by combining spinal cord-patterned neuronal and oligodendrocyte spheroids to replicate axon–glia interactions in vitro. Using a microfluidic platform, this system enables axonal fascicle formation, compact myelin deposition, and functional neuronal activity, offering a valuable framework for mechanistic research on fetal and perinatal white matter injury, including periventricular leukomalacia [233].

As discussed, vascularized (neurovascular) assembloids are generated by merging brain organoids with vascular organoids or mesodermal cell spheroids. This enables endothelial cells to infiltrate brain organoids and form vessel-like networks resembling capillaries, thereby dramatically improving organoid health, reducing the number of hypoxic cores, and supporting the growth of microglia and astrocytes that rely on vascular signals [232]. For perinatal injury models, vascularized organoids are a game changer; they can be used to better model hypoxic-ischemic injury, where blood vessels themselves are part of the pathology. A recent study used cortical organoid–blood vessel assembloids to examine the effects of SARS-CoV-2 on the fetal brain. The assembloid showed that viral exposure led to neuroinflammation and even Alzheimer-like amyloid plaque features in the organoid, which were mediated by the introduced endothelial cells and microglia [225]. This implies a synergy between infection and vascular injury that might contribute to neuropathology, which can only be observed in a vascularized brain model.

Future directions include placenta–brain assembloids: By coculturing or fusing placental/trophoblast organoids with brain organoids in a microfluidic or organ-chip system, one can simulate the maternal–fetal interface and explore the effects on fetal brain development, which are relevant to maternal infection, inflammation, toxin exposure, or placental dysfunction that impact perinatal brain injury and preterm birth [234]. Trophoblast organoids derived from human trophoblast stem cells recapitulate key placental villus features, including the syncytiotrophoblast barrier and vascular interactions [235]. More recently, a vascularized placental organoid microphysiological system (MPS) has been developed to model placental barrier function and immune responses to viral infection. These models are the essential groundwork for studying placental effects on the fetal brain [234].

### Microfluidic/brain organoid-on-chip systems

Brain organoid-on-chip platforms have recently gained recognition as a rapidly advancing and promising field that integrates organoid culture with microfluidic technologies to increase the physiological relevance of culture conditions. These systems optimize the organoid microenvironment by reducing cellular exposure to shear stress, increasing the oxygen supply and distribution, promoting chemical gradients, and improving control over parameters such as cell‒cell and cell‒extracellular matrix interactions, cell type composition, and tissue architecture. Furthermore, their dynamic culture conditions, with continuous nutrient infusion and waste removal, increase intracellular exchange, limit organoid core necrosis [148], and provide the physiological, biochemical, and physical cues necessary for brain development, thereby supporting long-term survival and the establishment of complex cytoarchitecture [232, 236, 237]. A recent study described a microfluidic system for midbrain organoid culture in which a peristaltic pump provided continuous laminar flow of medium with a flow rate comparable to physiological interstitial flow [238], thereby promoting neural differentiation and synaptic extension. Compared with conventional static culture, this approach improved oxygen penetration into the organoid core, reduced necrosis, and enhanced the differentiation of neural stem cells into dopaminergic neurons [239].

### Sexual dimorphism and genetic background considerations

An important but often overlooked factor in both disease modeling and drug testing is biological diversity, encompassing differences between males and females, as well as between individuals with different genetic backgrounds. Brain organoids offer opportunities to incorporate this diversity in experimental designs. Many perinatal brain injuries have sex-specific outcomes. Notably, male infants are more vulnerable to ischemic insult and display greater long-term neurodevelopmental deficits than females [131, 240, 241]. Furthermore, several studies have indicated that male offspring exposed to maternal drug abuse *in utero* are more vulnerable than females to dysfunctions in cognitive processing and emotional regulation [242]. Since most studies either pool male and female outcomes or limit analyses to one sex, the mechanisms driving sex differences remain incompletely understood. Experimental findings in rodent models of hypoxia-ischemia-inflammation (HII) support the male disadvantage in brain injury, potentially linked to male-specific heightened inflammatory responses, such as greater peripheral lymphocyte infiltration and increased levels of cytokines, including TNF-α. These differential inflammatory responses may contribute to the so-called “female advantage” observed in neonates [241, 243]. To capture such differences in vitro, organoids can be generated from male and female iPSC lines, where intrinsic sex chromosome effects (XX vs. XY) may influence developmental trajectories and disease phenotypes [244]. Brain organoids can also be treated with sex hormones such as estrogen and testosterone to determine how they modulate injury responses [244].

Every iPSC line carries the genetic and epigenetic legacy of the individual from whom it was derived. This includes common polymorphisms and rare variants that might affect neurodevelopment or drug metabolism. In a recent study, Anton-Bolanos et al. developed a “brain chimeroid”—a multidonor organoid model generated from multiple donor cell lines—to investigate the effects of ethanol and the antiseizure drug valproic acid (VPA). This approach enables the creation of organoids that capture interindividual variability in susceptibility to neurotoxic stimuli. Exposure of these chimeroids to VPA resulted in an increase in immature interneurons and choroid plexus cells, accompanied by a reduction in intermediate progenitors, whereas ethanol exposure led to a decrease in callosal projection neurons [245]. Interestingly, gene expression changes vary among donor lines; for example, one donor exhibited heightened sensitivity to ethanol but resilience to VPA, whereas the gene expression changes induced by VPA differed across donors. This variability could be due to differences in the alleles of genes involved in the metabolism of substances, baseline developmental gene expression, or different epigenetic mechanisms. This application of chimeroids is an appealing approach to determine interindividual variation in the processes of brain development and disease.

**Fig. 4:**
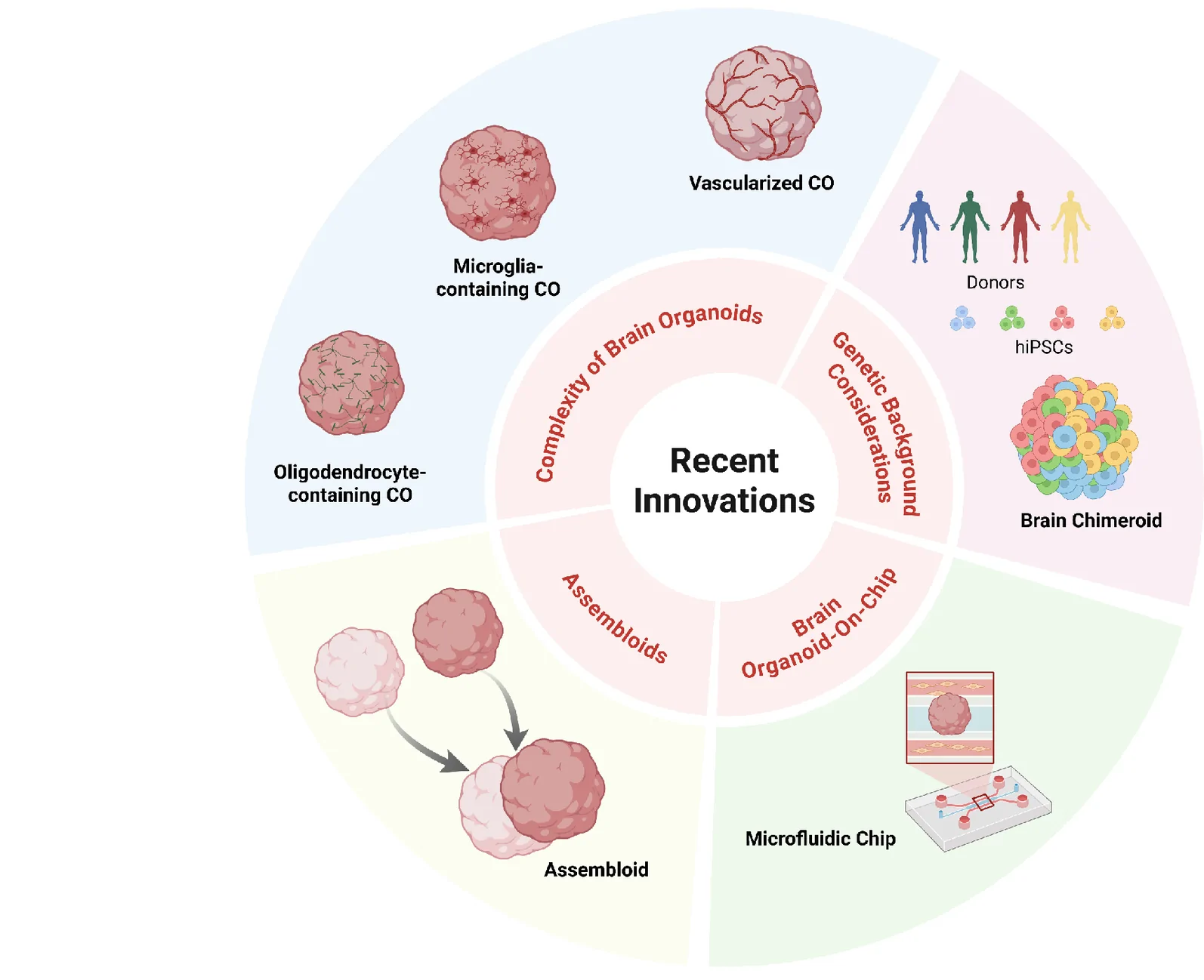
Recent innovations to improve the physiological relevance of brain organoids for modeling PBI. Created in https://BioRender.com

To account for genetic variability, researchers now often use multiple iPSC lines in organoid studies (3–4 lines from different donors). In the context of PBI, genetic background is crucial because conditions such as intrauterine inflammation or perinatal stroke do not affect all infants equally; some infants are resilient, whereas others are severely impacted [246, 247]. Organoid models can include patient-derived lines from infants who have a certain exposure but different outcomes to better understand protective vs. risk-associated cellular phenotypes. Ultimately, the diversity of organoid studies will make findings more clinically translatable. It also moves the field toward precision medicine, where therapies might be tailored on the basis of an infant’s sex or genetic profile, informed by how their cells respond in vitro. Thus, personalized therapeutics have the potential to account for interindividual variability and diverse patient phenotypes, enabling tailored diagnosis and more accurate prognostication [248, 249] (Fig. 4).

## Conclusion and future directions

Despite progress in brain organoid technology, some technical and translational challenges remain. Although human brain organoids closely recapitulate key aspects of neocortical development, their application for modeling perinatal insults still demands careful validation. In vivo, the fetal brain is protected by multiple barriers that are absent in vitro, including maternal detoxification of xenobiotics, the placental barrier, and the fetal blood‒brain barrier. The placenta, in particular, serves a dual function by transporting maternal hormones, nutrients, and oxygen to the fetus while simultaneously protecting it from xenobiotics through selective transport, enzymatic inactivation, and efflux mechanisms. These processes restrict the entry of large or water-soluble molecules unless specific transporters are present. Furthermore, whether a compound crosses the blood‒brain barrier remains an additional critical consideration. Another important limitation of standard organoid cultures to bear in mind is the absence of peripheral immune components, which contribute substantially to the pathogenesis of perinatal brain injury [250]. This challenge is further complicated by the fact that immune cells present in fetal and neonatal circulation differ substantially in maturation state and function from those of adults [250], limiting the utility of simply coculturing organoids with immune cells obtained from mature donors. Thus, these limitations highlight that current single-organoid models cannot fully capture the systemic distribution of drugs and metabolites within the body or whole-body inflammatory responses [13, 251]. The emergence of multiorganoid- and body-on-chip technologies, in which multiple organoids are embedded within a single microfluidic device and interconnected through channel networks designed to simulate human blood circulation, enables the study of interorgan interactions and provides a more holistic and physiologically relevant model for evaluating drug efficacy [252]. Furthermore, next-generation bioengineered platforms, known as quaternary chips, bring together complex region-specific organoids; integrate microfluidic networks that provide physiologically relevant perfusion and mechanical stimuli; embed biosensors capable of tracking metabolic, electrical, and molecular dynamics in real time; and use biohybrid components such as synthetic vascular structures and immune cell niches to replicate systemic interactions [253].

Collectively, brain organoids are increasingly recognized as transformative systems for modeling perinatal brain injury and neurodevelopmental disorders. They combine the strengths of human relevance with the flexibility of cell culture, enabling both basic science (elucidating mechanisms of injury) and translational research (evaluating drug candidates). However, findings from organoid models require rigorous validation through complementary approaches, such as animal studies, patient-derived data, and computational simulations, to confirm their biological relevance and translational applicability.

## Data Availability

No datasets were generated or analysed during the current study.

## Abbreviations

2D: Two-dimensional
3D: Three-dimensional
ACE2: Angiotensin-converting enzyme 2
AMPA: α-Amino-3-hydroxy-5-methyl-4-isoxazolepropionic acid
AQP1: Aquaporin-1
ASD: Autism spectrum disorder
ATP: Adenosine triphosphate
AXL: AXL receptor tyrosine kinase
BBB: Blood-brain barrier
BMP: Bone morphogenetic protein
BMSCSec: Bone marrow stem cell secretome
CB1: Cannabinoid receptor type 1
Cd: Cadmium
ChP: Choroid plexus
CMV: Cytomegalovirus
CNS: Central nervous system
CO: Cerebral organoid
CP: Cerebral palsy
CXCL9: C-X-C motif chemokine ligand 9
DOR: δ-Opioid receptor
DPM: Diesel particulate matter
DPP4: Dipeptidyl peptidase-4
EB: Embryoid body
EC: Endothelial cell
ECM: Extracellular matrix
E/I: Excitatory/inhibitory
FASD: Fetal alcohol spectrum disorders
GC: Glucocorticoid
GR: Glucocorticoid receptor
GW: Gestational week
HI: Hypoxia-ischemia
HIE: Hypoxic-ischemic encephalopathy
hPSC: Human pluripotent stem cell
HSV: Herpes simplex virus
HSV-1: Herpes simplex virus type 1
HSV-2: Herpes simplex virus type 2
IFN-γ: Interferon-gamma
IL-4: Interleukin-4
IL-6: Interleukin-6
IL-8: Interleukin-8
IL-10: Interleukin-10
IP: Intermediate progenitor cell
iPSCs: Induced pluripotent stem cells
ISSCR: International Society for Stem Cell Research
IZ: Intermediate zone
JEV: Japanese encephalitis virus
KOR: κ-Opioid receptor
MAT: Medication-assisted treatment
MEA: Microelectrode array
MHC-I: Major histocompatibility complex class I
Mpox: Monkeypox virus
MOR: µ-Opioid receptor
MSC: Mesenchymal stromal cell
NMDA: N-Methyl-D-aspartate
NOP: Nociceptin/orphanin FQ peptide receptor
NPC: Neural progenitor cell
NSC: Neural stem cell
NTD: Neural tube defect
OGD: Oxygen-glucose deprivation
OL: Oligodendrocyte
OPC: Oligodendrocyte progenitor cell
OUD: Opioid use disorder
PBI: Perinatal brain injury
Pb: Lead
pmOL: Premyelinating oligodendrocyte
POE: Prenatal opioid exposure
PSC: Pluripotent stem cell
PTB: Preterm birth
RG: Radial glia
ROS: Reactive oxygen species
SARS-CoV-2: Severe acute respiratory syndrome coronavirus 2
scRNA-seq: Single-cell RNA sequencing
SVZ: Subventricular zone
THC: Δ9-Tetrahydrocannabinol
TLR3: Toll-like receptor 3
TNF-α: Tumor necrosis factor-alpha
TMPRSS2: Transmembrane serine protease 2
UPR: Unfolded protein response
vCO: Vascularized cerebral organoid
VEGF: Vascular endothelial growth factor
VO: Vascular organoid
VZ: Ventricular zone
WMI: White matter injury
WNV: West Nile virus
ZIKV: Zika virus
